# Does arthroscopic patellar denervation with high tibial osteotomy improve anterior knee pain?

**DOI:** 10.1186/s40634-021-00411-5

**Published:** 2021-11-09

**Authors:** Mohamed Kamel Mohamed Said, Hatem G. Said, Hesham Elkady, Mahmoud Kamel Mohamed Said, Islam Karam-Allah Ramadan, Mohamed Abd EL-Radi

**Affiliations:** Faculty of Medicine, Assiut University, Assiut, 71515 Egypt

**Keywords:** Arthroscopic patellar denervation, Anterior knee pain, Patellofemoral, High tibial osteotomy, Tibiofemoral, Osteoarthritis

## Abstract

**Purpose:**

Patellofemoral (PF) joint osteoarthritis (OA) is a major cause of anterior knee pain. Combined PF and medial tibiofemoral (TF) OA is common in older adults. We evaluated the effect of arthroscopic patellar denervation (PD) in patients with combined TF and PFOA after malalignment correction.

**Methods:**

Forty-five patients [females/males, 27/18; age, 30–59 years (45.5 ± 8.50); mean body mass index, 25.15 ± 3.04 kg/m^2^] were treated in our department from March 2017 to March 2019. The patients were randomised into 2 groups: group A included 22 patients who underwent open-wedge high tibial osteotomy (OWHTO) and arthroscopic PD and group B included 23 patients who underwent OWHTO without denervation. The effect of denervation was statistically and clinically evaluated using the Knee injury and Osteoarthritis Outcome Score (KOOS) and Kujala (anterior knee pain score) score.

**Results:**

After 24 months, 40 patients were available for the final follow-up. The final values of KOOS and the Kujala score were significantly different between the groups (*p* < 0.001). For group A, the average KOOS improved from 42.73 to 72.38 (*p* < 0.001) and the Kujala score improved from 42 to 74.1 (*p* < 0.001), whereas in group B, the average KOOS improved from 39.22 to 56.84 (*p* < 0.001) and the Kujala score improved from 39.7 to 56.4 (*p* < 0.001).

**Conclusion:**

Adding arthroscopic PD to OWHTO relieves anterior knee pain in patients with combined TF and PFOA and improves knee joint function and quality of life.

**Level of evidence:**

Level I prospective randomised control clinical trial.

## Introduction

Patellofemoral (PF) osteoarthritis (OA) is a highly prevalent disease and an important cause of anterior knee pain. It greatly affects several daily activities, including kneeling, squatting, climbing stairs and getting up from a low chair [20, 22]. PFOA represents 60% of symptomatic knee OA, whereas combined PF and tibiofemoral (TF)OA (40%) is a more common form of knee OA than PFOA (24%) or TFOA alone (4%) [16, 17]. PF pain occurs in approximately 7.3% of the patients in the United States [13]. Boling et al. [5] demonstrated that anterior knee pain primarily affects middle-aged people and is 2.23-fold more common in females than males (our random sample size is consistent with these findings; 24 females and 16 males) [12].

Knee pain and disability are more severe in combined PF and TFOA, which is a common presentation in older adults [17]. Open-wedge high tibial osteotomy (OWHTO) is commonly used for treating medial compartment TFOA with varus knees [30]. Although OWHTO has shown good clinical results in patients with medial knee OA, this procedure is not recommended for patients with severe PFOA [30]. OWHTO may decrease patellar height (patella baja) [10, 36], which increases contact stress on the PF joint and eventually leads to anterior knee pain [21, 45]. Therefore, arthroscopic patellar denervation (PD), a joint-preserving minimally invasive technique, may provide relief for anterior knee pain and decrease the limitations of OWHTO.

Research has shown that the incidence of anterior knee pain following total knee arthroplasty can be reduced by PD [51]. Vega et al. [48] described the technique for arthroscopic PD and its effect on anterior knee pain. However, to our knowledge, no studies have investigated the addition of arthroscopic PD to OWHTO in patients with combined PF and TFOA. Therefore, the present study assessed anterior knee pain and complications after OWHTO with or without arthroscopic PD in patients with combined PF and TFOA.

## Methods

### Patient selection

**Fig. 1:**
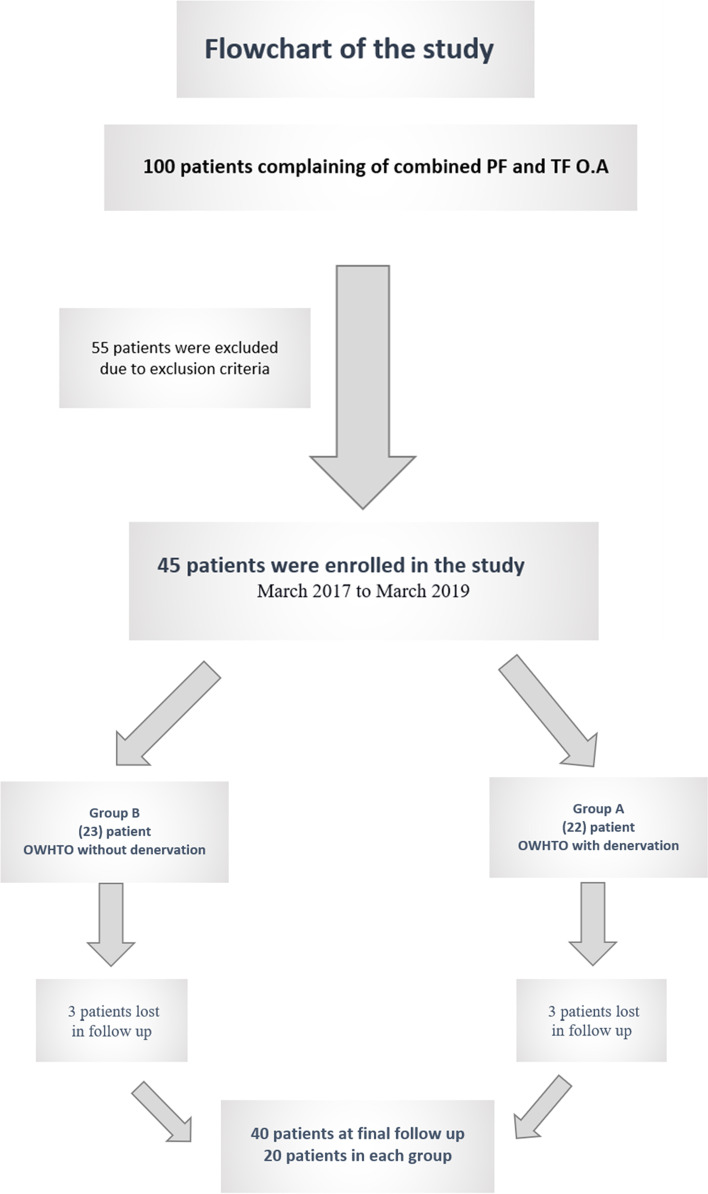
Patient flow chart

A prospective randomised control clinical trial was conducted in the arthroscopy unit of Assiut University Hospital. Forty-five patients [females/males, 27/18; age, 30–59 years (45.5 ± 8.50); mean body mass index (BMI), 25.15 ± 3.04 kg/m^2^] from March 2017 to March 2019 were enrolled in this study (Fig. 1).

The inclusion criteria were as follows: 1) age 20–60 years, 2) anterior knee pain (all grades of PFOA), 3) mild to moderate TFOA [G I–III Kellgren–Lawrence (KL) scale], 4) no involvement of the lateral compartment, 5) range of motion of at least 120° flexion and 6) BMI < 30 kg/m^2^. The exclusion criteria were as follows: 1) advanced case of TFOA (G VI KL scale), which requires total knee replacement; 2) flexion deformity ≥15°; 3) varus angle degree > 10° and 4) inflammatory disease (such as rheumatoid arthritis).

The patients were randomised into two groups using a sealed envelope system. Both groups (A and B) underwent OWHTO. Group A underwent OWHTO with arthroscopic PD, whereas group B underwent OWHTO without denervation. The effect of denervation was statistically and clinically evaluated using KOOS and the Kujala score. Diagnostic arthroscopy was performed in all cases to assess PF articular cartilage degeneration, classify the cartilage defect using the Outerbridge classification [42], assess the lateral compartment and exclude any other pathology.

### Clinical assessment

All patients were diagnosed using clinical history, physical examination and radiological assessment. They were then evaluated using a scoring system for patellofemoral disorders, which included the Kujala score [27] and KOOS [39]. The average duration of complaints was 30 months (6 months–5 years). The clinical manifestations included anterior knee pain, recurrent knee swelling, sense of friction and crepitus during flexion and extension of the knee, significant difficulty in removing socks, positive patellar grinding test and atrophy of the quadriceps femoris. Pain was primarily located at the patellar edges and was aggravated by climbing stairs, kneeling, standing from a seated position and squatting. All patients received conservative treatment (mean duration, 4 months) before surgical intervention, including the strengthening of the quadriceps muscle, lifestyle modification, analgesics and non-steroidal anti-inflammatory drugs, which were unsuccessful.

### Radiological assessment

#### X-ray anteroposterior view (Fig. 2)

The 5-point KL scale [25] was used to detect the severity of knee osteoarthritis, including mild to moderate grades (I, II, III) and excluding severe TFOA grades (G VI).

**Fig. 2:**
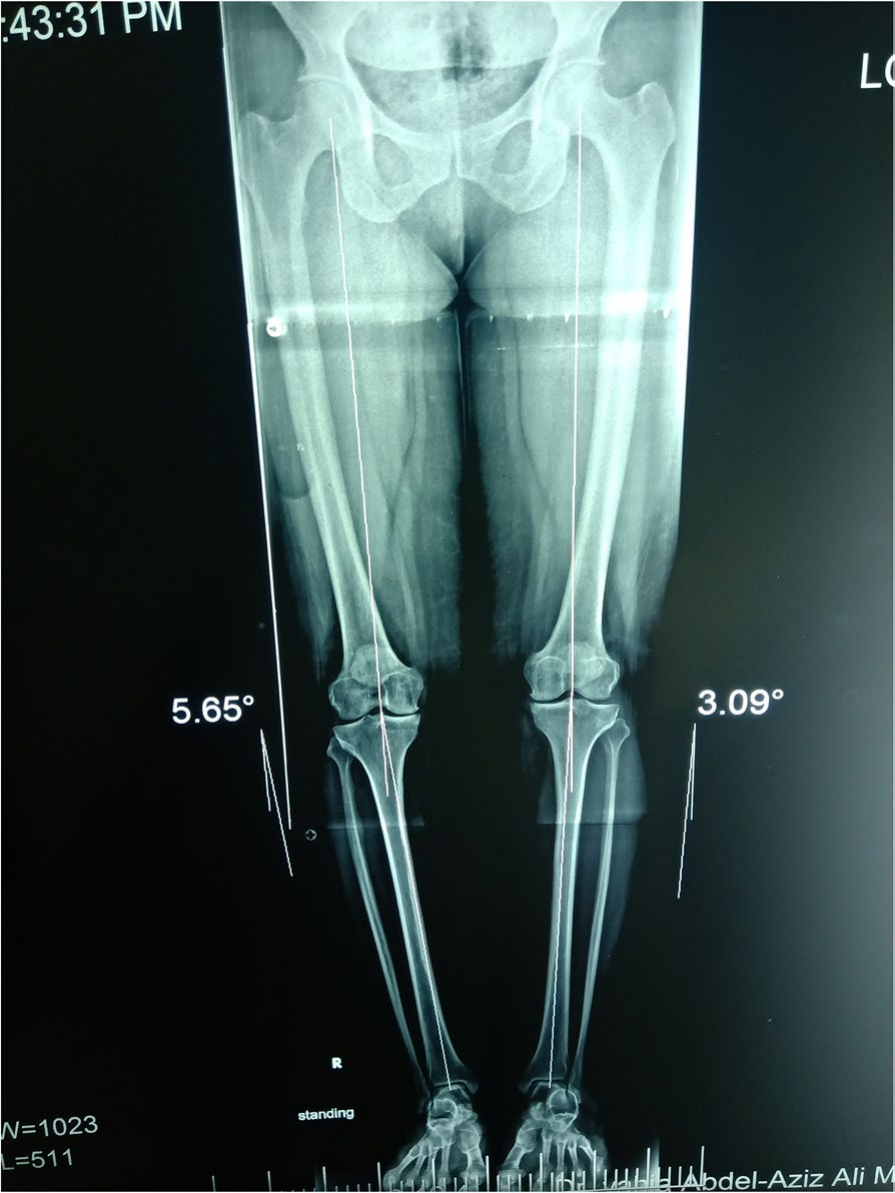
Anteroposterior long standing film shows bilateral varus knee with tibiofemoral osteoarthritis

#### The full-length standing radiograph in the anteroposterior view

This was used to assess the mechanical axis and exclude varus deformity of > 10° (Fig. 2).

#### The knee skyline Merchant 45° view

**Fig. 3:**
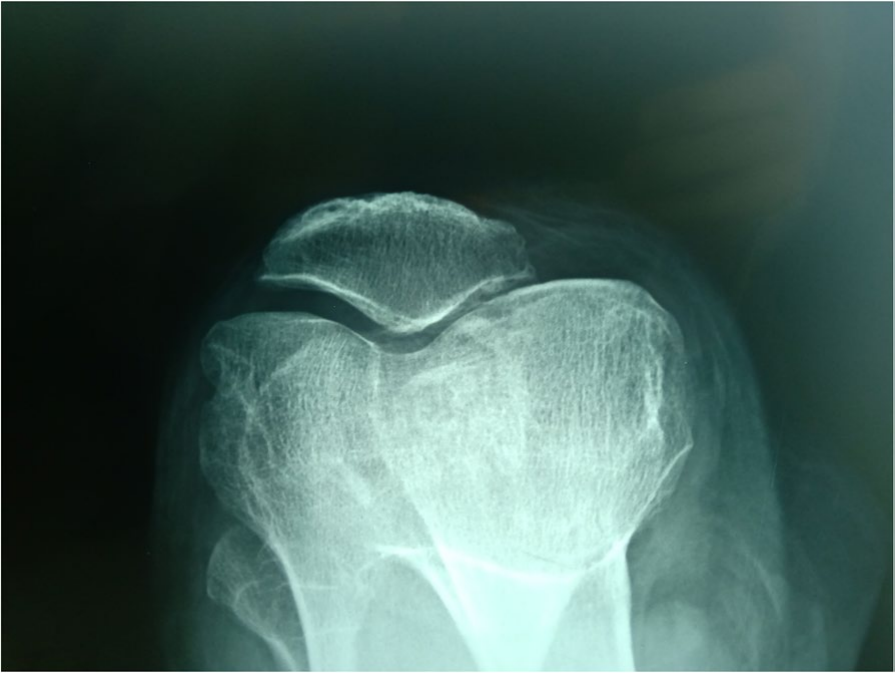
Patellofemoral joint osteoarthritis in the skyline view. The patellofemoral joints are degenerated, and the space between the patellofemoral joints is narrow

**Fig. 4:**
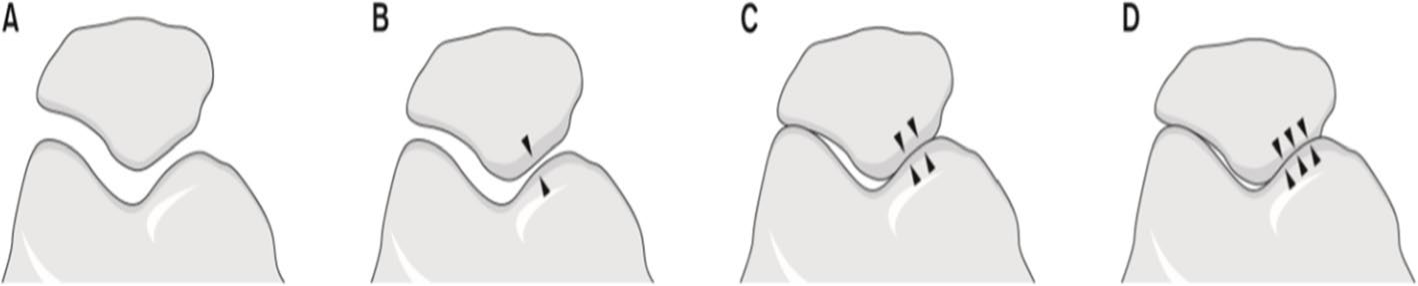
Merchant staging of the PFOA severity based on the 45° skyline view

This showed that the PF joint was degenerated and the space between the PF joint was narrow. PF joint arthritis was classified into four stages based on the 45° skyline view according to Merchant classification (Figs. 3 and 4) [32].

#### Lateral x-ray

Patellar height (alta or baja) may be evaluated by various methods in the lateral x-ray. We used the Insall–Salvati ratio with a normal ratio of 0.8–1.2. This compared the length of the patellar tendon with the patellar height [49].

### Arthroscopic PD technique

**Fig. 5:**
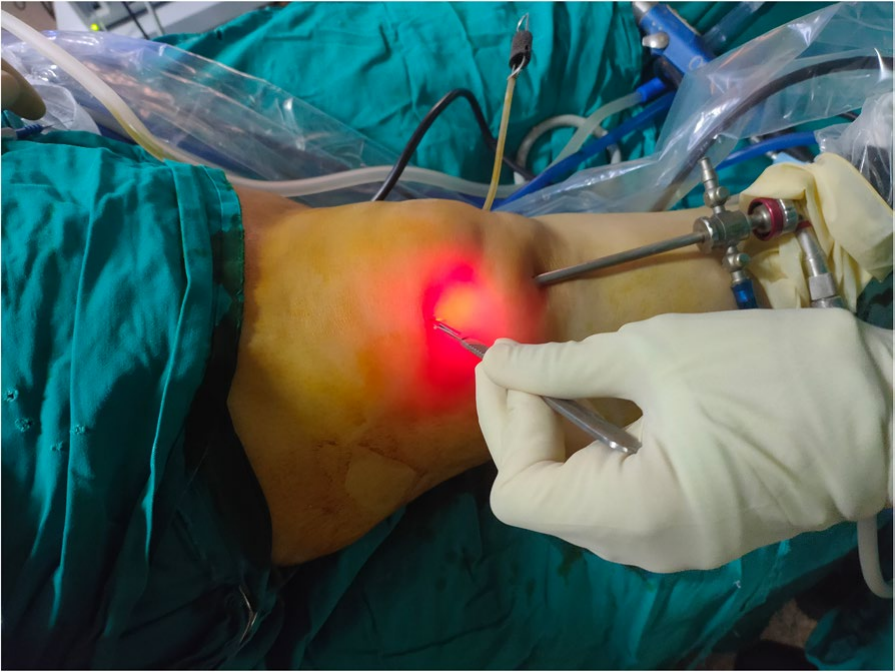
Knee suprapatellar portals

**Fig. 6:**
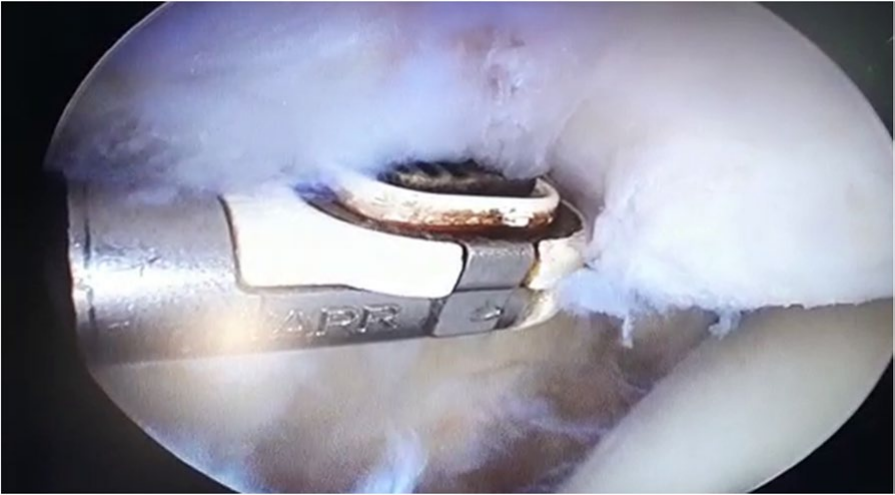
Arthroscopic patellar denervation technique

With the leg extended (Fig. 5), an arthroscope was inserted through the anteromedial, anterolateral and suprapatellar portals to access the entire perimeter of the patella. Next, the hyperplastic synovium in the intercondylar fossa was cleared and resected of plica (if present), and shaving, debridement and abrasive chondroplasty were subsequently performed. The patella was then denervated under vision using VAPER [Mitek VAPR3 3 Electrosurgical ESU Radiofrequency Unit 225,021 (9038), DePuy Mitek, Inc.] (Fig. 6). Conventional VAPER produces a thermal lesion to the peripatellar soft tissue; this obliterates a considerable number of pain receptors.

We used the technique described by Vega et al. [48] who recommended that denervation should not include the region of the patellar tendon. This was based on Scapinelli’s study [41] that showed that the aforementioned region is an important site of entry for blood vessels that reach the patella. Injury to these vessels could lead to patellar necrosis [50]; thus, we were cautious during denervation at the site of the patellar tendon insertion. Vega et al. [48] considered that the risk of complications resulting from patellar vascular injury is very low even though a partial disruption of the patellar vascularisation occurs and that neither the deep vessels nor the course of the vessels through the patellar tendon are affected. The arthroscopic denervation technique does not result in complete denervation and the proprioception and slight sensation of pain is preserved according to Vega et al. [48]. Therefore, the technique probably does not result in neurogenic arthropathy, which leads to patellofemoral arthrosis.

**Fig. 7:**
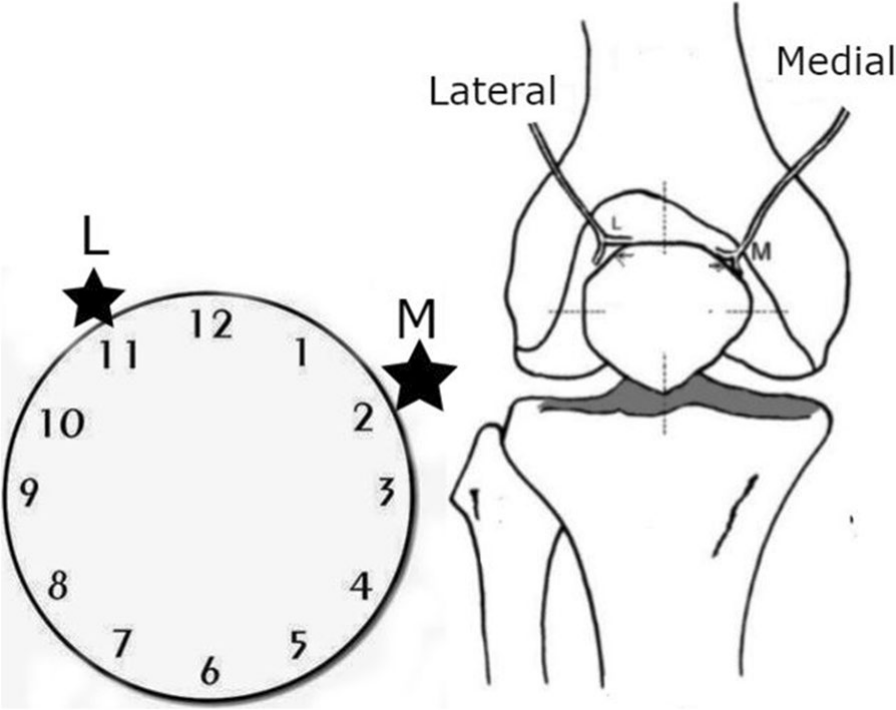
Entry point of the patellar nerves on the right knee

With respect to the patellar nerve supply, the two main nerves reach the superomedial and superolateral quadrants and emerge from vastus medialis and lateralis, entering the patella at 11:00 and 2:00 in a clockwise direction, respectively (Fig. 7) [31]. Research has demonstrated an anatomical variant to this innervation [50]. Because of the wide anatomical variability, selective neurotomy does not result in complete PD in many cases [48, 50]. In addition, some immunohistochemical studies reported hyperinnervation of the peripatellar soft tissue in patients with anterior knee pain, which contain a considerable number of nociceptors [3, 40, 50]. Based on these findings, we performed denervation on the peripatellar tissue and focused on the common entry sites of the supplying patellar nerves at 11:00 and 2:00.

### OWHTO surgical technique

**Fig. 8:**
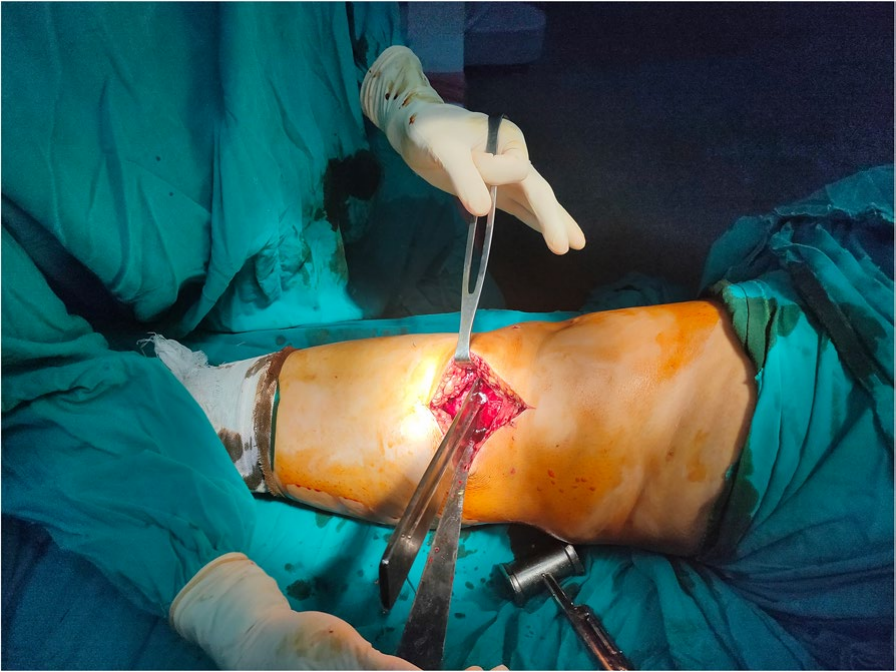
Open-wedge high tibial osteotomy after denervation

After arthroscopic denervation, all patients underwent OWHTO (Fig. 8). All procedures were performed based on the technique recommended by the AO international knee expert group. Biplanar osteotomy, which comprises osteotomies in the axial and frontal planes, was performed in all cases. Ascending frontal osteotomy, leaving the tibial tuberosity on the distal fragment, was also performed. All osteotomies were performed without the use of additional bone grafts, and the opening of the osteotomy was maintained with a T-locked plate (Orthomed-E Co., Egypt). Suction was inserted into the lateral arthroscopic portal, and wound closure was performed in layers.

### Postoperative rehabilitation

**Fig. 9:**
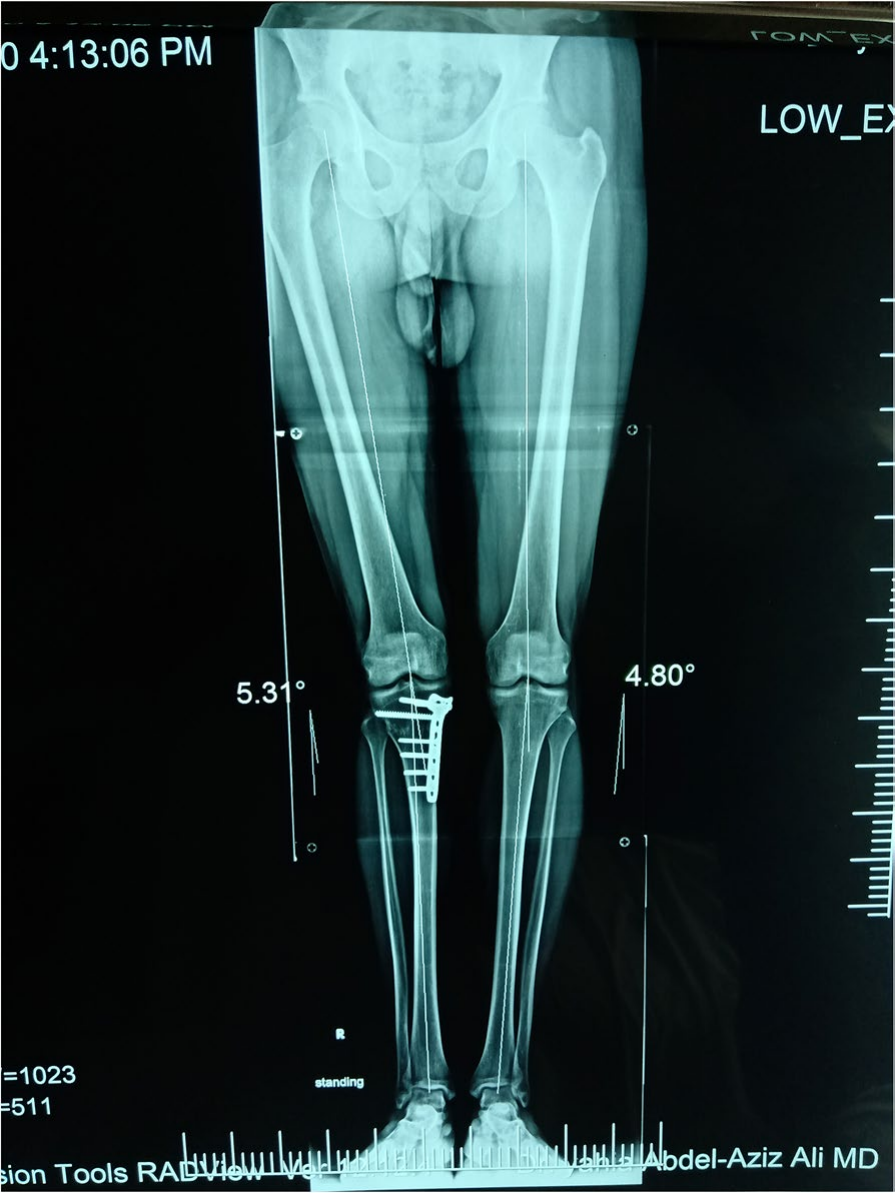
Postoperative long standing film

**Fig. 10:**
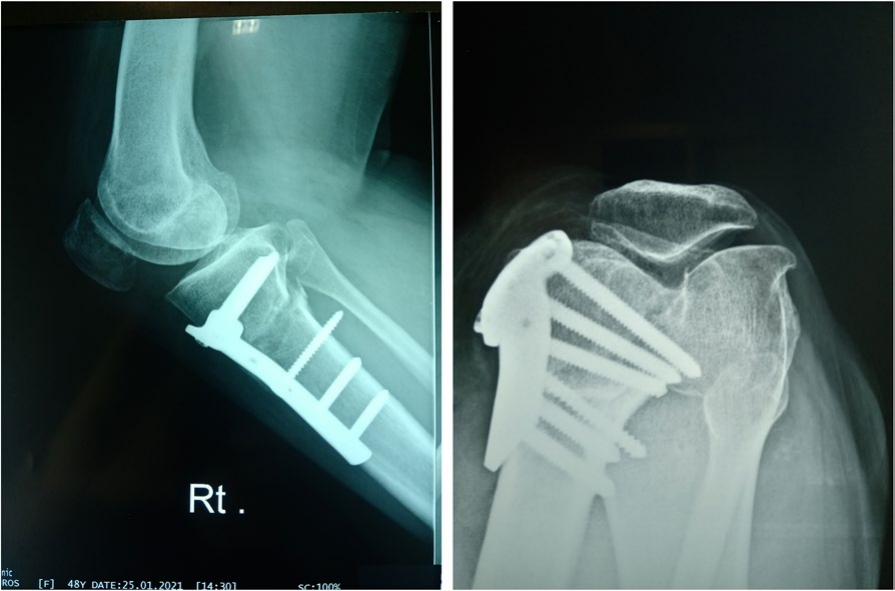
Postoperative x-ray: skyline and lateral views

Immediately after the procedure, all patients began isometric quadricep contractions and active ankle exercises. From day 3 to 2 weeks after the procedure, the patients performed exercises that included range of motion exercises, straight leg raising, active knee flexion and extension, isometric quadriceps contractions and stretching exercises for the hamstring, gastrocnemius and soleus. They also underwent ambulation with two crutches without weight bearing on the affected lower limb. At 6 weeks after the procedure, the patients continued home exercises and started partial weight-bearing ambulation with two crutches after an x-ray to detect healing (Figs. 9 and 10).

### Postoperative follow-up

All patients were evaluated during the follow-up period for 2 years (3rd, 6th, 12th, 18th and 24th months) using KOOS and the Kujala score to evaluate the therapeutic effects and improvements in knee joint function after surgery. The minimal clinically important difference (MCID) [9] was determined via a distribution-based method (the minimal change was approximately half a standard deviation of baseline scores) [33, 34].

### Statistical analyses

SPSS version 23.0 software was used for data management and analyses. The mean ± standard deviation with medians and ranges, when appropriate, were used to describe the quantitative data. The sample size was determined via a power analysis (considering an alpha error of 0.05 and power of 90%, a minimum sample size of 28 (14 patient for each group) was required for a moderately strong correlation). However, the sample size was increased to 45. Numbers with percentages were used to describe the qualitative data. The Chi-square test and Fisher’s exact test were used for comparing independent categorical variables. Where continuous data were normally distributed, Student’s t-test was used for comparisons between two groups and repeated measures analysis of variance. For non-normally distributed data, the Mann–Whitney and Kruskal–Wallis tests were used. The significance level was set at α = 0.05. *P* < 0.05 was considered a significant difference.

## Results

Forty-five patients met the inclusion criteria clinically and radiologically and five patients were lost to follow-up. Forty patients were available for final evaluation (endpoint, follow-up of 2 years) (Fig. 1). All enrolled patients completed the questionnaires during their pre- and postoperative follow-up. There were no complications such as infection, nerve or vascular injury or ischaemic necrosis, although there were two cases, one in each group, of superficial surgical site infection at the site of the incision, who were treated with broad-spectrum antibiotics.

**Fig. 11:**
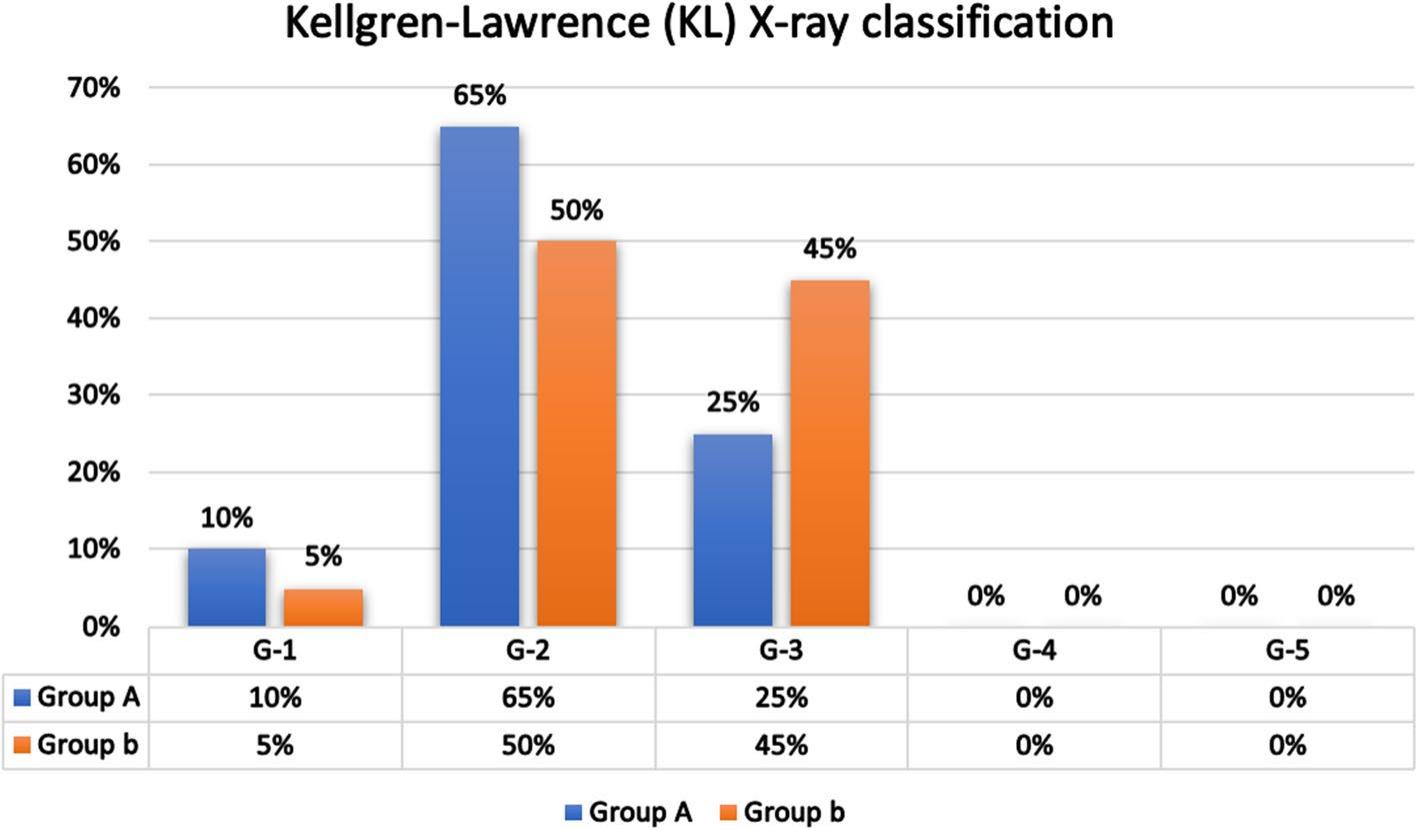
Preoperative Kellgren–Lawrence scale of both groups

**Fig. 12:**
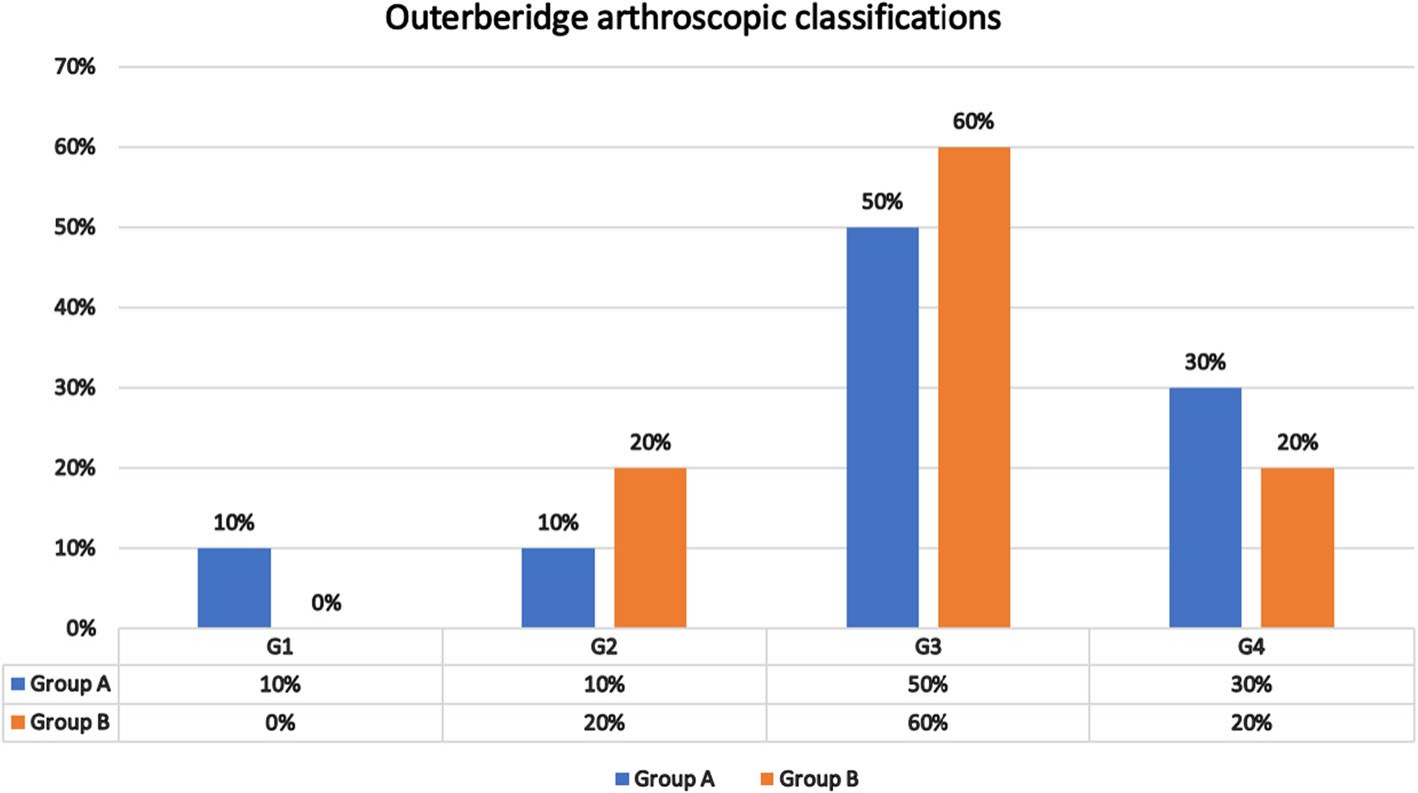
Preoperative Outerbridge arthroscopic classifications of both groups

**Fig. 13:**
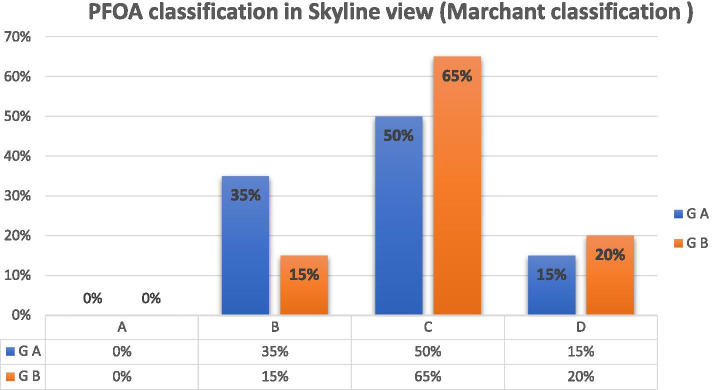
Preoperative patellofemoral joint osteoarthritis classification in the skyline view x-rays (Merchant classification) of both groups

**Fig. 14:**
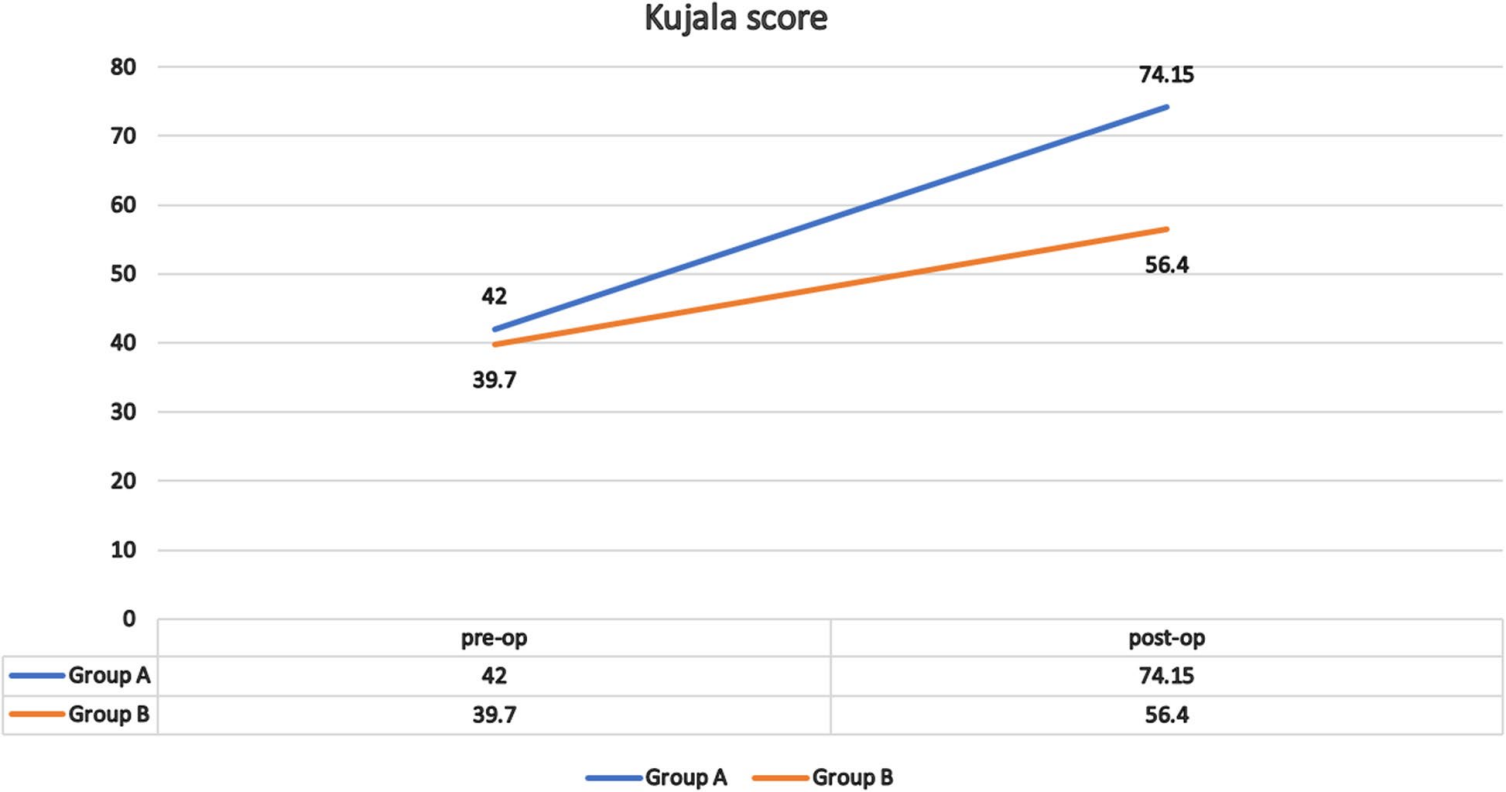
Pre- and postoperative KUJALA scores with % difference from baseline in both groups

**Fig. 15:**
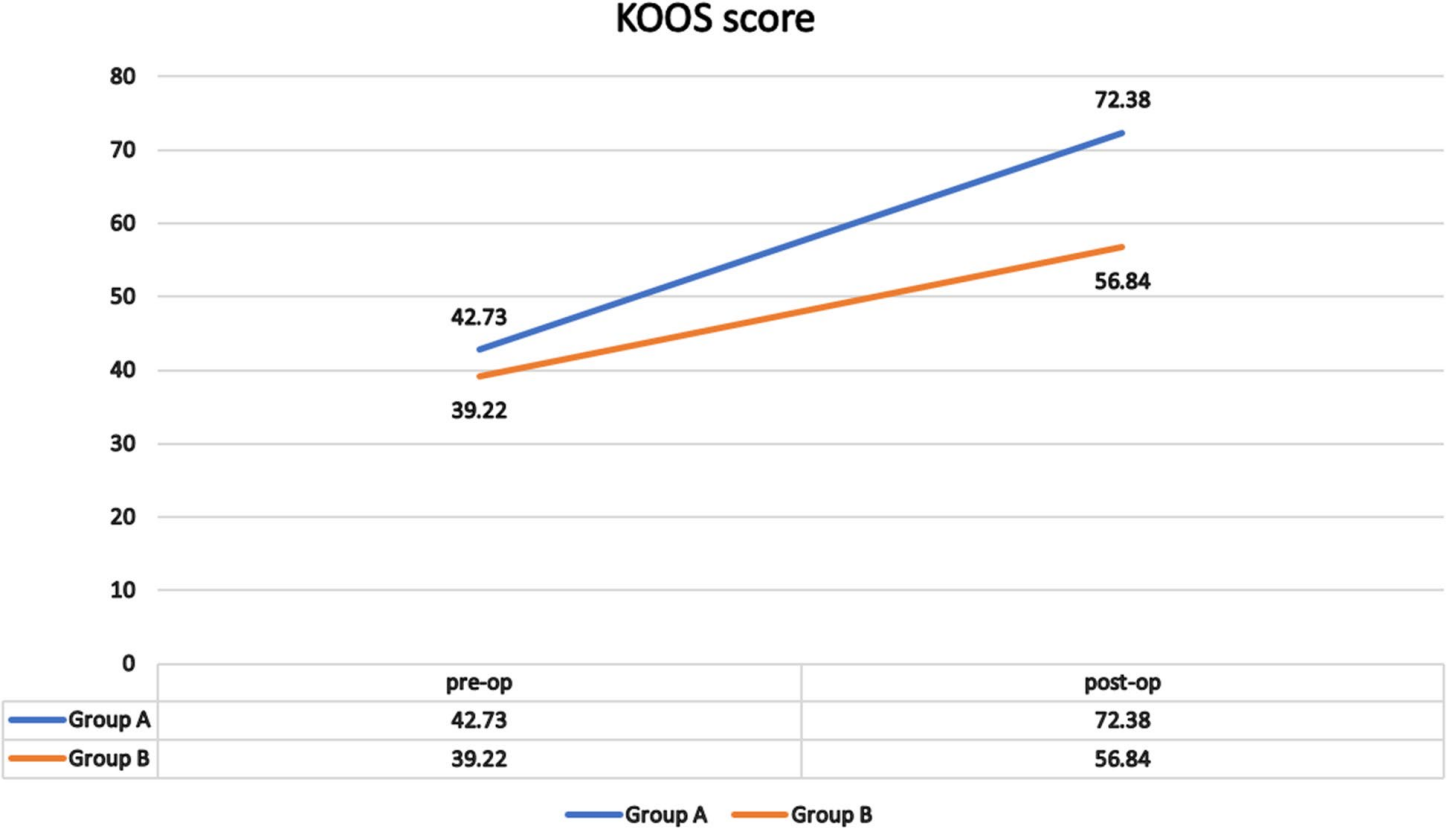
Pre- and postoperative KOOS with % difference from baseline in both groups

At the preoperative assessment, there were no epidemiological, clinical, radiological (anteroposterior x-ray KL classification and skyline Merchant classification), arthroscopic (Outerbridge classification) or the Kujala score and KOOS differences between the two groups (Tables 1, 2 and 3; Figs. 11, 12 and 13). However, both groups showed a significant improvement statistically and clinically according to KOOS and the Kujala score (*p* < 0.001); group A improved significantly more than group B (*p* < 0.001) (Tables 4 and 5). For group A, the average KOOS improved from 42.73 to 72.38 (*p* < 0.001) and the Kujala score improved from 42 to 74.1 (*p* < 0.001). For group B, the average KOOS improved from 39.22 to 56.84 and the Kujala score improved from 39.7 to 56.4 (*p* < 0.001) (Tables 4 and 5; Figs. 14 and 15).

**Table 1:** Mean age in both groups

Age (year)	Group A	Group B	***P***-value
**Mean**	45.45	45.65	0.862 (NS)
**SD +/−**	8.50	9.3	
**Median**	45	44.5	
**Range**	30-58	28-59

**Table 2:** Mean MBI in both groups

		Study groups	
		Group A	Group B
**BMI (kg/m2)**	**Mean**	26.12	24.12
	**±SD**	2.10	3.04
	**Median**	26	23.50
	**Range**	23-29	18.5-29

**Table 3:** X-ray Kellgren-Lawrence (KL) classification of knee OA in both groups pre-operative

X-ray K.L classification	Group A		Group B		***P***-value
Degree of O. A	count	%	count	%	
**Grade 0**	0	0	0	0	.472 (NS)
**Grade 1**	2	10	1	5	
**Grade 2**	13	65	10	50	
**Grade 3**	5	25	9	45	
**Grade 4**	0	0	0	0	
**Total**	20	100	20	100

**Table 4:** Kujala score before treatment, after treatment with % difference from baseline in both groups

Study group	Kujala score pre-op		Kujala score post-op		Kujala % increase		***P*** value for time effect
	Mean	SD	Mean	SD	Mean	SD	
**Group A**	42.00	11.42	74.15	8.12	84.25	35.51	**< 0.001**
**Group B**	39.70	9.93	56.40	10.55	46.41	30.04	**< 0.001**
**SD both**	10.63 (MCID)						
***P*value between groups**	1.0		**< 0.001**		**0.006**

**Table 5:** KOOS score before treatment, after treatment with % difference from baseline in both groups

Study group	KOOS score pre-op		KOOS score post-op		KOOS % increase		***P*** value for time effect
	Mean	SD	Mean	SD	Mean	SD	
**Group A**	42.73	9.22	72.38	7.11	74.97	32.56	**< 0.001**
**Group B**	39.22	8.85	56.84	7.78	49.37	25.46	**< 0.001**
**SD both**	9.09 (MCID)						
***P*value between groups**	1.0		**< 0.001**		**0.078**

MCID was calculated via a distribution-based method (approximate half a standard deviation of the baseline scores) using the total scores of the initial visits from all the participants; MCID was 5 points for the Kujala score and 4.5 points for KOOS (Tables 4 and 5).

The mean degree of varus malalignment for group A was 5.432 and for group B was 6.887. After OWHTO, the postoperative mean degree of varus malalignment was − 0.490 and 0.852, respectively (Table 6). Moreover, there was no significant difference in the degree of correction between the two groups.

**Table 6:** Degree of varus malalignment before treatment and after treatment in both groups

Study groups	Degree of Varus pre-op		Degree of Varus post-op		*P* value for time effect
	Mean	SD	Mean	SD	
Group A	5.432	2.901	−0.490	2.928	< 0.001*
Group B	6.887	1.699	0.852	1.754	< 0.001*
*P* value	0.432		0.508		

OWHTO significantly corrected varus deformity in both study groups (p < 0.001). But the degree of correction was not different among both groups

*p* value is significant ≤0.05, all *p* values are Bonferroni adjusted

The correlation between the TF x-ray classification (KL) and postoperative outcome [Kujala score (*p* = 0.069) and KOOS (*p* = 0.063)] (Table 7) was negative and borderline statistically significant. This indicates that an increase in the degree of TFOA by x-ray KL classification is associated with a decrease in the Kujala score and KOOS in both groups.

**Table 7:** Correlation between Kellgren-Lawrence (KL) classification of knee OA and outcome in both groups

Item (Spearman’s rho)	X-ray K.L classification	
	*r*- value	*p*- value
**KOOS score for both groups**	−0.297	0.063
**Kujala score for both groups**	−.0291	0.069

The correlation between x-ray classification and outcome (post-op scores for Kujala and KOOS) was fair negative association but borderline statistically significant

Correlation is significant at the 0.05 level (2-tailed)

The correlation between age and postoperative outcome [Kujala score (*p* = 0.016) and KOOS (*p* = 0.039)] (Table 8) was negative and statistically significant. This means that increased age (years) is associated with a decrease in the Kujala score and KOOS in both groups. Patellar height, as measured by the Insall–Salvati index, was significantly decreased after OWHTO (*p* = 0.006) (Table 9).

**Table 8:** Correlation between age and outcome both groups

Item (Pearson Correlation)	Age(year)	
	***R***-value	***P***-value
**KOOS sore post-op (both groups)**	− 0.328	0.039
**Kujala sore post-op (both groups)**	−0.380	0.016

The correlation between age and outcome (post-op scores for Kujala and KOOS) was fair negative statistically significant

Correlation is significant at the 0.05 level (2-tailed)

**Table 9:** Difference in Insall-Salvati index in both groups pre-operative

Both groups	Mean	SD	***P***-value
**Insall-Salvati pre-op (*N*** **= 40)**	1.1320	0.21681	0.006
**Insall-Salvati post-op (*N*** **= 40)**	1.0488	0.23901	

*p* value is significant ≤0.05, all *p* values are Bonferroni adjusted

Although, adding arthroscopic PD to OWHTO significantly improved KOOS and the Kujala score in group A, old age and severe grades of TFOA were associated with less favourable outcomes. All PFOA stages (radiological or arthroscopic) improved to the same degree with no significant difference among PFOA degrees. Regarding this result, we believe that arthroscopic PD outcome does not depend on the preoperative degree of PFOA (radiologically or arthroscopically) (Tables 10 and 11).

**Table 10:** Correlation between Merchant classification (skyline view) of PFJ O.A and outcome in group A

Group A	Merchant classification (skyline view) of PFJ O. A								***P***-value
PFOA grades	A		B		C		D		
	Mean	SD	Mean	SD	Mean	SD	Mean	SD	(NS)
**Kujala pre-op**			44.1	14.2	41.6	11.1	38.3	6.4	0.972
**Kujala post-op**			77.6	8.2	71.8	8.0	74.0	8.0	0.373
**KOOS pre-op**			45.3	9.8	42.3	9.7	38.2	6.3	0.504
**KOOS post-op**			74.4	8.6	71.5	7.2	70.4	1.6	0.641

**Table 11:** Correlation between Outerbridge arthroscopic classification of PFJ O.A and outcome in group A

Group A	Outerbridge arthroscopic classification of PFJ								***P***-value
PFOA grades	1		2		3		4		
	Mean	SD	Mean	SD	Mean	SD	Mean	SD	(NS)
**Kujala pre-op**	36.5	3.5	61.0	19.8	41.3	10.0	38.7	8.0	0.264
**Kujala post-op**	74.0	4.2	81.0	15.6	74.4	8.0	71.5	7.4	0.593
**KOOS pre-op**	42.6	1.3	52.7	18.9	41.8	7.9	41.0	9.7	0.483
**KOOS post-op**	72.9	9.7	75.1	12.7	73.3	8.3	69.8	2.4	0.850

## Discussion

The main finding of the present study was that arthroscopic PD improves anterior knee pain, and this technique should be considered in patients with combined medial TF and PFOA undergoing OWHTO. These results confirm the hypothesis that patients with anterior knee pain and medial TFOA with varus knee will benefit greatly from arthroscopic PD after malalignment correction. Therefore, we designed a prospective randomised clinical trial to test this hypothesis by comparing two groups of patients. Group A comprised 22 patients who underwent OWHTO and arthroscopic PD, whereas group B included 23 patients who underwent OWHTO without denervation. Both groups improved statistically and clinically according to KOOS and the Kujala score (*p* < 0.001); however, group A exhibited significantly higher scores than group B (*p* < 0.001).

MCID is an important concept used to determine whether a medical intervention improves perceived outcomes in patients [9]. Three different methods are used to calculate MCID: the distribution-based method, anchor-based method and Delphi method [33]. Norman et al. [34] proposed the standard deviation method and reported that in patients with chronic disease, the estimates of minimal change were approximately half a standard deviation of the baseline scores. It was calculated using the total scores of the initial visits from all participants and was 5 points for the Kujala score and 4.5 points for KOOS. Using the anchor-based method, Çelik et al. [9] determined the MCID of KOOS and the Kujala score (both scores ranged from 0 to 100). KOOS (MCID) was 14.5 [9], whereas the Kujala (MCID) score was 9.5. Jacquet et al. [19] determined the MCID values of KOOS for patients who underwent OWHTO and the values were 15.4 for KOOS pain, 15.1 for KOOS symptoms, 17 for KOOS ADL, 11.2 for KOOS sports/reaction and 16.5 for KOOS QQL.

According to the present study, in group A, the improvement in KOOS was 32.56 and the Kujala score was 32.15, whereas in group B, the improvement in KOOS was 25.46 and the Kujala score was 16.7. Improvement in both groups was greater for MCID calculated using the two methods, but group A was superior to group B. In group B, both scores in some patients were lower than expected after OWHTO. This decrease may be explained by the culture and lifestyle in our locality. The patients included farmers and manual workers who are used to continuous squatting and stair climbing during their work, and they showed less satisfaction, particularly in activities related to the PF joint (kneeling, squatting and climbing stairs).

Another solution for combined medial TF and PFOA is unicompartmental knee arthroplasty (UKA) plus PD. Although UKA shows good clinical results in patients with medial knee OA, it is not recommended for patients with severe PFOA. Suwankomonkul et al. [44] conducted a prospective comparative study in which UKA plus PD via circumferential electrocautery were performed. This study demonstrated that PD decreases short-term anterior knee pain in patients with PFOA undergoing UKA. The Kujala score after 6 months was approximately 10 points higher than that observed after UKA without PD. Even patients with high-grade full-thickness cartilage loss of the patella (grade III–IV) showed significant improvement in anterior knee pain scores compared with patients without denervation.

According to Zhao et al. [52], 156 PFOA patients with intact tibiofemoral (males/females, 62/94; age, 45–81 years, mean, 66 years) were treated with arthroscopic patelloplasty, lateral retinaculum release (LRR) and arthroscopic PD. The therapeutic effects of surgery significantly improved both the Lysholm and Kujala scores (*P* < 0.05). The authors [52] claimed that arthroscopic PD is closely associated with the degree of PF articular cartilage degeneration. Improvement was limited to stage I–III cases and was not observed among patients with cartilage defect IV. In contrast to the results of Zhao et al. [52], the present study showed that all stages of PFOA (radiological or arthroscopic) exhibited improvement to the same degree with no significant difference. Based on this result, we suggest that arthroscopic PD outcome is not dependent on the degree of PFOA. There are several possible explanations for this finding. First, the study focused on patients that were up to 81 years old. Most patients complained of severe grades of PFOA, and LRR was performed on all patients. Tao et al. [46] conducted a study on 60 cases with anterior knee pain, which were recruited for arthroscopic PD and intra-articular patelloplasty with extra-articular retinaculum release. The Feller patellar score [11] was used rather than the Kujala score and only patients with x-ray KL classification (0, 1, 2) were included. At the last follow-up, there was a significant difference between the scores before and after surgery (*P* ≤ 0.05). In contrast to the present study, LRR was performed in all patients; the use of LRR in relieving anterior knee pain is a matter of debate. It has significant limitations and specific indications [1, 29, 38] and may yield less favourable outcomes in some cases. Our results showed that old age and severe TFOA grades were associated with a less favourable outcome after OWHTO plus arthroscopic PD. An increase in age (years) and the degree of TFOA is associated with a decrease in the postoperative Kujala score and KOOS.

Some studies have reported an effect of age on outcome after OWHTO. The ISAKOS Congress in 2005 defined the ideal age for patients undergoing OWHTO as 40–60 years [7]. Kohn et al. [24], Goshima et al. [14] and Floerkemeier et al. [12] reported that age does not affect clinical and radiological outcomes after OWHTO. By contrast, Holden et al. [15] and Odenbring et al. [35] showed better results in patients aged < 50 years. Moreover, Trieb et al. [47] reported that the failure rate after OWHTO was significantly higher in patients aged > 65 years than in younger patients. Bonasia et al. [6] found that the risk of unsuccessful surgery was 5-fold higher in patients aged > 56 years.

With respect to the correlation between the degree of TFOA and postoperative outcome, Bonasia et al. [6] found better outcomes in patients with low grade medial compartment arthritis. By contrast, Floerkemeier et al. [12] reported good outcomes even in patients with severe mono-compartmental arthritis (G III or IV) OA.

In the present study, patellar height, measured via the Insall–Salvati index, significantly decreased after OWHTO (*p* = 0.006). Several studies [2, 4, 8, 10, 28] have reported a decrease in postoperative patellar height after OWHTO, which strongly depends on the degree of the correction angle, particularly in patients requiring major axis correction [2]. Amzallag et al. [2] recommended the routine baseline measurement of patellar height before OWHTO in patients requiring major axis correction. In addition, El-Azab et al. [10] recommended shifting to closed-wedge osteotomy in patients with borderline patella baja.

The effect of OWHTO on patellar height can be avoided by minor modifications to biplanar osteotomy using a descending limb rather than an ascending limb procedure. Krause et al. [26] and Kloos et al. [23] conducted a comparative study between ascending and descending biplanar OWHTO. The results showed that only descending OWHTO preserved patellar height, whereas ascending OWHTO resulted in a significant increase in patellar height, causing a significant increase in the PF joint contact pressure and anterior knee pain [23, 26].

Varus knee deformity is associated with worsening PFOA [18, 37]. OWHTO is commonly used for treating arthritic medial compartment with varus knee [30]. Some studies have reported the adverse effects of OWHTO on the PF joint [21, 43, 45] and progression of patellofemoral cartilage degeneration after OWHTO with large alignment correction. Song et al. [43] and Tanaka et al. [45] recommended a careful consideration of the OA status of the PF joint and the required correction angle. Further, it is well known that advanced PFOA is a contraindication for OWHTO [30]. Based on these considerations, we suggest that adding arthroscopic PD to OWHTO may relieve any pre-existing anterior knee pain and protect against intermediate term complications (such as anterior knee pain with progressive cartilage degeneration). Thus, arthroscopic PD decreases the limitation of OWHTO and delays joint replacement surgery.

There are certain limitations to this study. First, this technique is a symptomatic rather than a curative treatment method and cannot repair or alter PFOA. Second, 24 months is a relatively short follow-up period. A longer follow-up period is needed to validate the clinical results of arthroscopic PD. Finally, a larger sample size is needed to firmly recommend the method.

## Conclusions

To our knowledge, this is the first study to evaluate the effect of adding arthroscopic PD to OWHTO in patients with combined medial TF and PFOA. Patients with anterior knee pain caused by PFOA with varus knee may benefit greatly from arthroscopic PD after malalignment correction. This technique can reduce the severity and incidence of anterior knee pain, improve the quality of life, enhance daily activity and delay total knee arthroplasty.

## Abbreviations

KL: Kellgren–Lawrence
KOOS: Knee injury and Osteoarthritis Outcome Score
LRR: Lateral retinaculum release
MCID: Minimal clinically important difference
OWHTO: Open-wedge high tibial osteotomy
PD: Patellar denervation
UKA: Unicompartmental knee arthroplasty

## References

[1] Alemdaroğlu KB, Çimen O, Aydoğan NH, Atlıhan D, İltar S (2008). Early results of arthroscopic lateral retinacular release in patellofemoral osteoarthritis. Knee.

[2] Amzallag J, Pujol N, Maqdes A, Beaufils P, Judet T, Catonne Y (2013). Patellar height modification after high tibial osteotomy by either medial opening-wedge or lateral closing-wedge osteotomies. Knee Surg Sports Traumatol Arthrosc.

[3] Biedert RM, Sanchis-Alfonso V (2002). Sources of anterior knee pain. Clin J Sport Med.

[4] Bin S-I, Kim H-J, Ahn H-S, Rim DS, Lee D-H (2016). Changes in patellar height after opening wedge and closing wedge high tibial osteotomy: a meta-analysis. Arthroscopy.

[5] Boling M, Padua D, Marshall S, Guskiewicz K, Pyne S, Beutler A (2010). Gender differences in the incidence and prevalence of patellofemoral pain syndrome. Scand J Med Sci Sports.

[6] Bonasia DE, Governale G, Spolaore S, Rossi R, Amendola A (2014). High tibial osteotomy. Curr Rev Musculoskelet Med.

[7] Brinkman J-M, Lobenhoffer P, Agneskirchner J, Staubli A, Wymenga A, Van Heerwaarden R (2008). Osteotomies around the knee: patient selection, stability of fixation and bone healing in high tibial osteotomies. J Bone Joint Surg Br.

[8] Brouwer R, Bierma-Zeinstra S, Van Koeveringe A, Verhaar J (2005). Patellar height and the inclination of the tibial plateau after high tibial osteotomy: the open versus the closed-wedge technique. J Bone Joint Surg Br.

[9] Celik D, Çoban Ö, Kılıçoğlu Ö (2019). Minimal clinically important difference of commonly used hip-, knee-, foot-, and ankle-specific questionnaires: a systematic review. J Clin Epidemiol.

[10] El-Azab H, Glabgly P, Paul J, Imhoff AB, Hinterwimmer S (2010). Patellar height and posterior tibial slope after open-and closed-wedge high tibial osteotomy: a radiological study on 100 patients. Am J Sports Med.

[11] Feller JA, Bartlett RJ, Lang DM (1996). Patellar resurfacing versus retention in total knee arthroplasty. J Bone Joint Surg Br.

[12] Floerkemeier S, Staubli AE, Schroeter S, Goldhahn S, Lobenhoffer P (2013). Outcome after high tibial open-wedge osteotomy: a retrospective evaluation of 533 patients. Knee Surg Sports Traumatol Arthrosc.

[13] Glaviano NR, Kew M, Hart JM, Saliba S (2015). Demographic and epidemiological trends in patellofemoral pain. J Orthop Sports Phys.

[14] Goshima K, Sawaguchi T, Sakagoshi D, Shigemoto K, Hatsuchi Y, Akahane M (2017). Age does not affect the clinical and radiological outcomes after open-wedge high tibial osteotomy. Knee Surg Sports Traumatol Arthrosc.

[15] Holden DL, James S, Larson R, Slocum D (1988). Proximal tibial osteotomy in patients who are fifty years old or less. A long-term follow-up study. J Bone Joint Surg Am.

[16] Iijima H, Fukutani N, Aoyama T, Fukumoto T, Uritani D, Kaneda E (2016). Clinical impact of coexisting patellofemoral osteoarthritis in Japanese patients with medial knee osteoarthritis. Arthritis Care Res (Hoboken).

[17] Iijima H, Fukutani N, Isho T, Yamamoto Y, Hiraoka M, Miyanobu K (2017). Changes in clinical symptoms and functional disability in patients with coexisting patellofemoral and tibiofemoral osteoarthritis: a 1-year prospective cohort study. BMC Musculoskelet Disord.

[18] Iijima H, Fukutani N, Yamamoto Y, Hiraoka M, Miyanobu K, Jinnouchi M (2017). Association of varus thrust with prevalent patellofemoral osteoarthritis: a cross-sectional study. Gait Posture.

[19] Jacquet C, Pioger C, Khakha R, Steltzlen C, Kley K, Pujol N (2021). Evaluation of the “Minimal Clinically Important Difference” (MCID) of the KOOS, KSS and SF-12 scores after open-wedge high tibial osteotomy. Knee Surg Sports Traumatol Arthrosc.

[20] Kobayashi S, Pappas E, Fransen M, Refshauge K, Simic M (2016). The prevalence of patellofemoral osteoarthritis: a systematic review and meta-analysis. Osteoarthr Cartil.

[21] Kim K-I, Kim D-K, Song S-J, Lee S-H, Bae D-K (2017). Medial open-wedge high tibial osteotomy may adversely affect the patellofemoral joint. Arthroscopy.

[22] Kim Y-M, Joo Y-B (2012). Patellofemoral osteoarthritis. Knee Surg Relat Res.

[23] Kloos F, Becher C, Fleischer B, Feucht MJ, Hohloch L, Südkamp N (2019). High tibial osteotomy increases patellofemoral pressure if adverted proximal, while open-wedge HTO with distal biplanar osteotomy discharges the patellofemoral joint: different open-wedge high tibial osteotomies compared to an extra-articular unloading device. Knee Surg Sports Traumatol Arthrosc.

[24] Kohn L, Sauerschnig M, Iskansar S, Lorenz S, Meidinger G, Imhoff A (2013). Age does not influence the clinical outcome after high tibial osteotomy. Knee Surg Sports Traumatol Arthrosc.

[25] Kohn MD, Sassoon AA, Fernando ND (2016). Classifications in brief: Kellgren-Lawrence classification of osteoarthritis. Clin Orthop.

[26] Krause M, Drenck TC, Korthaus A, Preiss A, Frosch K-H, Akoto R (2018). Patella height is not altered by descending medial open-wedge high tibial osteotomy (HTO) compared to ascending HTO. Knee Surg Sports Traumatol Arthrosc.

[27] Kujala UM, Jaakkola LH, Koskinen SK, Taimela S, Hurme M, Nelimarkka O (1993). Scoring of patellofemoral disorders. Arthroscopy.

[28] LaPrade RF, Barrera Oro F, Ziegler CG, Wijdicks CA, Walsh MP (2010). Patellar height and tibial slope after opening-wedge proximal tibial osteotomy: a prospective study. Am J Sports Med.

[29] Liu C, Duan G, Niu Y, Cao P, Fu K, Niu J (2018). Lateral retinaculum plasty instead of lateral retinacular release with concomitant medial patellofemoral ligament reconstruction can achieve better results for patellar dislocation. Knee Surg Sports Traumatol Arthrosc.

[30] Loia MC, Vanni S, Rosso F, Bonasia DE, Bruzzone M, Dettoni F (2016). High tibial osteotomy in varus knees: indications and limits. Joints.

[31] Maralcan G, Kuru I, Issi S, Esmer A, Tekdemir I, Evcik D (2005). The innervation of patella: anatomical and clinical study. Surg Radiol Anat.

[32] Merchant AC, Mercer RL, Jacobsen RH, Cool CR (1974). Roentgenographic analysis of patellofemoral congruence. J Bone Joint Surg Am.

[33] Mouelhi Y, Jouve E, Castelli C, Gentile S (2020). How is the minimal clinically important difference established in health-related quality of life instruments? Review of anchors and methods. Health Qual Life Outcomes.

[34] Norman GR, Sloan JA, Wyrwich KW (2003). Interpretation of changes in health-related quality of life: the remarkable universality of half a standard deviation. Med Care.

[35] Odenbring S, Tjörnstrand B, Egund N, Hagstedt B, Hovelius L, Lindstrand A (1989). Function after tibial osteotomy for medial gonarthrosis below aged 50 years. Acta Orthop Scand.

[36] Otsuki S, Murakami T, Okamoto Y, Nakagawa K, Okuno N, Wakama H (2018). Risk of patella baja after opening-wedge high tibial osteotomy. J Orthop Surg (Hong Kong).

[37] Otsuki S, Nakajima M, Okamoto Y, Oda S, Hoshiyama Y, Iida G (2016). Correlation between varus knee malalignment and patellofemoral osteoarthritis. Knee Surg Sports Traumatol Arthrosc.

[38] Panni AS, Tartarone M, Patricola A, Paxton EW, Fithian DC (2005). Long-term results of lateral retinacular release. Arthroscopy Knee Surg Relat Res.

[39] Roos EM, Lohmander LS (2003). The Knee injury and Osteoarthritis Outcome Score (KOOS): from joint injury to osteoarthritis. Health Qual Life Outcomes.

[40] Sanchis-Alfonso V, Roselló-Sastre E (2003). Anterior knee pain in the young patient-what causes the pain? “Neural model”. Acta Orthop Scand.

[41] Scapinelli R (1967). Blood supply of the human patella: its relation to ischaemic necrosis after fracture. J Bone Joint Surg Br.

[42] Slattery C, Kweon CY (2018). Classifications in brief: outerbridge classification of chondral lesions. Clin Orthop Relat Res.

[43] Song SJ, Yoon KH, Park CH (2020). Patellofemoral cartilage degeneration after closed-and open-wedge high tibial osteotomy with large alignment correction. Am J Sports Med.

[44] Suwankomonkul P, Arirachakaran A, Kongtharvonskul J (2020) Short-term improvement of patellofemoral pain in medial unicompartmental knee arthroplasty with patellar denervation: a prospective comparative study. Musculoskelet Surg:1–8. 10.1007/s12306-020-00675-7. 10.1007/s12306-020-00675-7 32743756

[45] Tanaka T, Matsushita T, Miyaji N, Ibaraki K, Nishida K, Oka S (2019). Deterioration of patellofemoral cartilage status after medial open-wedge high tibial osteotomy. Knee Surg Sports Traumatol Arthrosc.

[46] Tao J, Chen P, Chen J, Xie L, Liu L, Yang Y, Guo HM (2018). Arthroscopic patellofemoral denervation in the treatment of severe patellofemoral arthritis. Biomed Res.

[47] Trieb K, Grohs J, Hanslik-Schnabel B, Stulnig T, Panotopoulos J, Wanivenhaus A (2006). Age predicts outcome of high-tibial osteotomy. Knee Surg Sports Traumatol Arthrosc.

[48] Vega J, Golanó P, Pérez-Carro L (2006). Electrosurgical arthroscopic patellar denervation. Arthroscopy.

[49] Verhulst FV, van Sambeeck JD, Olthuis GS, van der Ree J, Koëter S (2020). Patellar height measurements: Insall–Salvati ratio is most reliable method. Knee Surg Sports Traumatol Arthrosc.

[50] Wojtys EM, Beaman DN, Glover RA, Janda D (1990). Innervation of the human knee joint by substance-P fibers. Arthroscopy.

[51] Xie X, Pei F, Huang Z, Tan Z, Yang Z, Kang P (2015). Does patellar denervation reduce post-operative anterior knee pain after total knee arthroplasty?. Knee Surg Sports Traumatol Arthrosc.

[52] Zhao G, Liu Y, Yuan B, Shen X, Qu F, Wang J (2015). Arthroscopic patelloplasty and circumpatellar denervation for the treatment of patellofemoral osteoarthritis. Chin Med J.

